# Generalized Anxiety Disorder and Its Associated Factors Among Pregnant Women During COVID-19 at Public Health Facilities of East Gojjam Zone, 2020: A Multi-Center Cross-Sectional Study

**DOI:** 10.3389/fgwh.2022.918332

**Published:** 2022-07-08

**Authors:** Keralem Anteneh Bishaw, Addisu Andalem, Haile Amha, Tirusew wondie

**Affiliations:** ^1^Department of Midwifery, College Health Science, Debre Markos University, Debre Markos, Ethiopia; ^2^Department of Nursing, College Health Science, Debre Markos University, Debre Markos, Ethiopia

**Keywords:** COVID-19, pregnant woman, generalized anxiety disorder, associated factors, Ethiopia

## Abstract

**Introduction:**

Pregnant women suffer from varying levels of generalized anxiety disorder that result in poor obstetrical outcomes. Therefore, this study aimed to assess the prevalence and factors associated with generalized anxiety disorder among pregnant women attending antenatal care during COVID-19 at the public health facilities in the east Gojjam zone.

**Methods:**

A health facility-based cross-sectional study was conducted, from 1–30 December 2020. A total of 847 pregnant women were included in the study using a systematic random sampling technique. We used an interviewer-administered questionnaire to collect the data. Bivariate and multivariable logistic regression was used to identify factors associated with the outcome variable. Statistical significance was determined using a *p*-value < 0.05 and a 95% confidence level.

**Results:**

The prevalence of generalized anxiety disorder was 43.7%, with a 95% CI (40.28–47.12). Having <3 the number of children (AOR: 1.53; 95% CI: 1.11–2.13, having a negative attitude about COVID (AOR: 1.47; 95% CI: 1.07–2.02 and having a high-risk perception about COVID (AOR: 1.86; 95% CI: 1.34–2.57 were factors significantly associated with generalized anxiety disorder.

**Conclusions:**

The study found that the prevalence of generalized anxiety disorder was high. Having less than three children, having a negative attitude, and having a high-risk perception of COVID were independent risk factors of generalized anxiety disorder. Appropriate interventions should be considered to address generalized anxiety disorder during the pandemic.

## Introduction

The 2019 coronavirus disease (COVID-19) pandemic is spreading at an accelerating rate (1) and has become an international concern of the world (2). Pregnant women and their fetuses are a high-risk population during pandemic infections. Physiological and mechanical changes during pregnancy generally increase susceptibility to infections and promote the rapid progression of pregnant women to respiratory failure if they are affected by the cardiopulmonary diseases (1). Even if pregnant women are highly susceptible, there are no specific drugs for COVID-19 that have not been found at present (3).

Infectious disease outbreaks can be distressing for everyone, especially for those particularly vulnerable groups, such as pregnant women (4), and associated with a high rate of anxiety among pregnant women (5). Pregnant women particularly need more attention (6) since they are highly susceptible to COVID-19, and the prognosis is worse after being infected when compared with the non-pregnant women (3). COVID-19 may predispose pregnant women to higher risks of severe disease and poorer neonatal outcomes (7). It is associated with serious psychological challenges for pregnant individuals, with the potential for both short-term (e.g., preterm birth, postpartum depression) and long-lasting impacts on the developing fetus (8).

The mental health of a pregnant mother is vital for preventing pregnancy and birth-related complications (9). The current uncertainties and alarming situation of the COVID-19 pandemic may cause anxiety, mental distress, and fears among pregnant women (10). Pregnancy can be stressful for women during normal times, yet the COVID-19 pandemic has amplified pregnancy-related anxiety (PRA) (11).

Anxiety during pregnancy is one of the most common mental health problems (12). PRA is a distinct type of anxiety that affects pregnant women and is characterized by pregnancy-specific fears and worries. It is associated with several bad obstetric, neonatal, and maternal outcomes (13). Pregnancy-related fears are pronounced in women with multiple anxiety disorders (14).

Pregnant women suffer from varying levels of anxiety that can negatively affect pregnancy outcomes (15). A study in Turkey reported an increased level of anxiety in pregnant women during the COVID-19 pandemic (16). A study in Iran showed that the prevalence of anxiety during COVID-19 was 21% (15). The pandemic is rapidly increasing in Ethiopia, and the number of perinatal service users at the hospital decreased due to the fear of contracting the virus (9). It induces a doubling of the number of women who experienced anxiety (17). The highest prevalence of antenatal anxiety is a serious public health concern since it is a significant risk factor for maternal morbidity and poor child health and development (18).

Because anxiety is common during pregnancy, identifying associated factors is pertinent for developing preventive measures during prenatal care (19). Despite the high rates of anxiety disorders among women reported across Africa before the outbreak of COVID-19, there was a noticeable absence of maternal mental health research in Africa during the epidemic (20). In addition, very limited evidence is available regarding antenatal anxiety and associated factors in Ethiopia. Therefore, this study aimed to assess the prevalence of GAD and risk factors among pregnant women attending antenatal care at public health facilities in the east Gojjam zone.

## Methods

### Study Area and Period

The study was conducted at the public health facilities of the east Gojjam zone of Amhara regional state from 1–30 December 2020. East Gojjam zone is a zone in the Amhara region of Ethiopia with a capital city of Debre Markos town (located 300 km from Addis Ababa, the capital city of Ethiopia, and 265 km from Bihar Dar, the capital city of Amhara). The East Gojjam zone has 19 districts and 468 kebeles. It also has ten (10) hospitals, 103 health centers, and 423 health posts. The zone has only one comprehensive specialized hospital (DMCSH).

### Study Design

A health facility based cross-sectional study was conducted.

### Population

The study's source population included all pregnant women at public health centers in the east Gojjam zone. The study included all the pregnant women who were available during the data collection period at selected public health facilities. Pregnant women with communication problems and/or women with critically ill and mentally impaired were excluded from the study.

### Sample Size Determination

The sample size was determined using single population proportion formula considering the following assumptions: proportion of GAD during COVID-19 in Ethiopia 50%, 95% confidence level, 5% margin of error (absolute level of precision). Thus, *n* = (Z_a/2_)^2^ $\frac{\;p\;\left(1-p\right)\;}{\left(d\right)\;2}=$ 1.96^2^^*^0.5^*^0.5/ (0.05)^2^ = 384.16~385. By considering a 10% non-response rate and design effect of 2, the final sample size of the study was 847.

### Sampling Procedure and Technique

**Figure 1:**
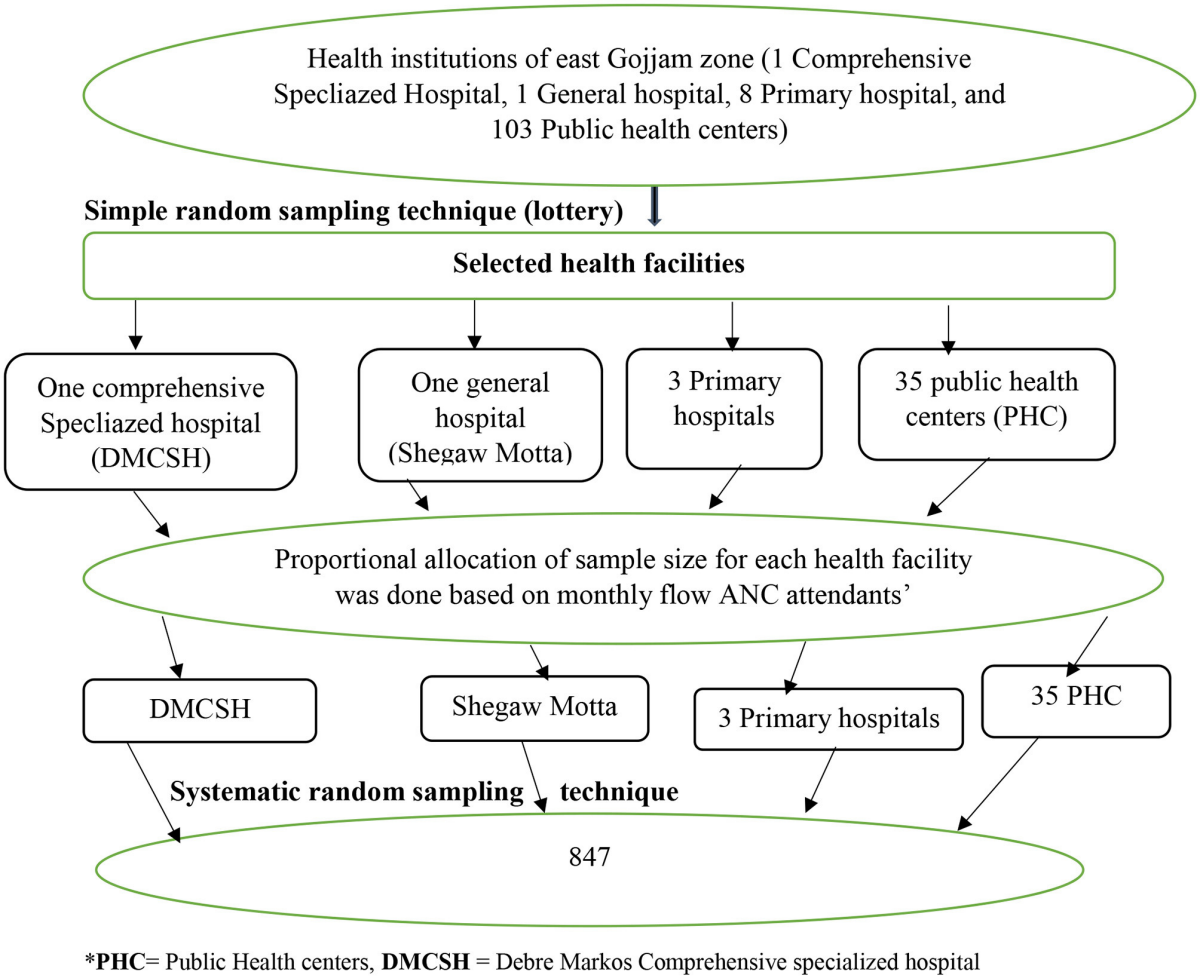
Diagrammatic presentation of the sampling procedure to assess generalized anxiety disorder and associated factors during COVID-19 pandemic among pregnant women attending antenatal care in East Gojjam zone public health facilities, 2020. PHC = Amber, Den, Wuseta, Mislewash, Kurar, Jamma, TsidMaryam, Wojel, Yesenbet, Waber, Gozamin, Yetnora, Weynwuha, Yelamgej, Debrework, Amba Maryam, Jeremis, Kuyy, Yebabat, Amanuel, Dega Segnin,Yekebabat, Debre-Elias, Gofichima, Genet, Kork, Guayi, Debre Markos, Angot, Sedie, Gundewoyn, Gietie Semanni, Girraram, Mergechi, Gemborie. Primary hospitals: Lumame, Bichena and Yejubie Primary Hospital.

A multistage sampling technique was used. First, stratification was done based on the level of health facility. Then, one-third (1/3) from each type of health facility was taken using a simple random lottery sampling technique. Then, the proportional allocation for each health facility was done to allocate the sample size. To sum, each study participant was selected using a systematic random lottery sampling technique (Figure 1).

### Study Variables

#### Dependent Variable

Generalized anxiety disorder during COVID-19.

#### Independent Variables

##### Socio-Demographic Characteristics

Age, residence, level of education, marital status, and occupation.

##### Obstetrical Characteristics

Gestational week of pregnancy, gravidity, and parity.

##### Individual Characteristics

Knowledge, attitude, practice, and risk perception of the pregnant woman about COVID-19.

### Operational Definition

#### Generalized Anxiety Disorders

It was diagnosed when a pregnant woman scored ≥7 on the Generalized Anxiety Disorder 7-item (GAD-7) questionnaire (21).

#### Adequate Knowledge

Pregnant women who scored greater than or equal to the mean value of 28 knowledge-related questions was considered as they had adequate knowledge about the COVID-19 pandemic.

#### Favorable Attitude

Pregnant women who scored greater than or equal to the mean value of ten attitude-related questions were considered as they had favorable attitude toward the COVID-19 pandemic.

#### Good Practice

Pregnant women who scored greater than or equal to the mean value of nine practice-related questions as they had good practice toward the COVID-19 pandemic prevention.

#### Risk Perception

Pregnant women who scored greater than or equal to the mean value of risk six perception-related questions as they had high-risk perception toward the COVID-19 pandemic prevention.

### Data Collection Tools and Procedures

Pretested and interviewer-administered questionnaires were used for the whole study. The questionnaire was adapted from the reviewed literature (11, 22–24) with modification and contextualized into the local setting. The questionnaire consisted of socio-demographic variables, obstetric history, knowledge, attitude, practice, and risk perception of pregnant women to the COVID-19 pandemic. The GAD-7 questionnaire is a 7-question scale used to measure pregnancy-related anxiety. It has four choices regarding the severity of symptoms: not at all, several days, more than half the days, and nearly every day, which correspond to 0, 1, 2, and 3 points score, respectively (minimum total score 0 and maximum 21). The total score of the GAD-7 ranges from 0 to 21. The total score of the GAD-7 ranges from 0 to 21. The questionnaire was drafted in English and translated into Amharic. The data were collected by trained BSc midwives and mental health professionals. The consistency and completeness of the data were checked daily by supervisors. A face mask was given to the participants and the data collectors during the data collection.

### Statistical Analysis

The consistency and completeness of the questionnaires were first checked manually. Then, the data were entered into a computer by Epi data 4.2.and exported SPSS 25 for analysis. Descriptive statistics were calculated to describe socio-demographic and other relevant variables. Data were presented using tables. Multivariable logistic regression carried for variables with *p*-value < 0.25 in bivariate logistic regression to identify factors. A *P*-value of <0.05 with a 95% confidence level was used to determine a statistical significance. This study followed a strobe statement reporting checklist of cross-sectional studies (25).

## Results

### Socio Demographic Characteristics

Out of the 847 sampled pregnant women, 806 responded to the questionnaires making a response rate of 95.2%. More than one-third (35.4%) of study participants were in the age group of 25–29 years old. The mean age of the study participants was 27.57 ± 6.08 years. Most of the study participants were predominately (94.6%), and more than 50 % (426) of study participants were married and urban residents, respectively. In total, three hundred forty-three (42.6%) of study participants did not attend formal education. More than 40% (329) of participants reported that their monthly income had decreased in the last 3 months (Table 1).

**Table 1:** Socio-demographic characteristics of pregnant women attending ANC during COVID-19 pandemic at public health facilities of East Gojjam zone, Amhara region, Ethiopia, 2020. (*n* = 806).

**Characteristics**	**Response**	**Frequency**	**Percent (%)**
Age	15–19	59	7.3
	20–24	199	24.7
	25–29	285	35.4
	30–34	130	16.1
	≥35	133	16.5
Marital status	Married	763	94.6
	Divorced	15	1.9
	Single	4	0.5
	Widowed	24	3
Residence	Rural	380	47.1
	Urban	426	52.9
Level of education	No formal education	343	42.6
	Primary	169	21
	Secondary	105	13
	Diploma and above*	189	23.4
Occupation	Housewife	498	61.8
	Civil servant	148	18.4
	Private business	103	12.8
	Others*	57	7
Monthly salary	≤1000	126	15.6
	1001–3000	386	47.9
	3001–10000	292	36.2
	>10000	2	0.2
Situation monthly income in the past three months	Worsened	329	40.8
	Improve	98	12.2
	Remain the same	379	47

Diploma and above*, BSc, MSc; Others*, daily laborer, farmer, student, and employed in private sector.

### Obstetrical History

This study reported that about 501 (62.2%) and 330 (40.9%) study participants were multigravidas and nulliparous, respectively. Regarding the status of abortion, about 70 (8.7%) had a history of abortion. Despite most of the study participants, 774 (96%) had wanted pregnancy, only 306 (38 %) had ≥ 3 ANC visits. In total, one hundred three (12.8%) study participants developed obstetrical complications. Of these, 27 (26.2%) and 19 (18.4%) developed hypertension and diabetes, respectively (Table 2).

**Table 2:** Obstetrical characteristics of pregnant women attending ANC during COVID-19 pandemic at public health facilities of East Gojjam zone, Amhara region, Ethiopia, 2020. (*n* = 806).

**Characteristics**	**Response**	**Frequency**	**Percent (%)**
Gravidity	Primi	305	37.8
	Multi	501	62.2
Parity	Nulliparous	330	40.9
	Primipara	170	21.1
	Multipara	306	38
Abortion	Yes	70	8.7
	No	736	91.3
Number of ANC visit	<3	500	62
	≥3	306	38
Condition of pregnancy	Wanted	774	96
	Unwanted	32	4
Gestational age	<37 weeks	739	91.7
	≥37 weeks	67	8.3
Number of alive children	<3 children	734	91.1
	≥3 children	72	8.9
Medical health problems	Yes	103	12.8
	No	703	87.2
Types of medical health problems (*N* = 103)	Hypertension	27	26.2
	Diabetes miltus	19	18.4
	Pneumonia	8	7.8
	Asthma	25	24.2
	Cardiac	5	4.9
	Tuberculosis	11	10.7
	HIV/AIDS	8	7.8

### Individual Characteristics of Study Participants

This study revealed that 412 (51.1%), 416 (51.6%), 300 (37.2%), and 354 (43.9%) pregnant women had a favorable attitude, adequate knowledge, high-risk perception, and good practice regarding the COVID-19 pandemic, respectively.

### Generalized Anxiety Disorder During COVID-19 Pandemic

The level of anxiety symptoms of pregnant women during the COVID-19 infection significantly increased. This study reported that the prevalence of GAD during the COVID-19 pandemic was 43.7 % (95% CI; 40.28–47.12).

### Factors Associated With Generalized Anxiety Disorder

In bivariate logistic regression living in the urban area, educational level, number of alive children, attitude, knowledge, risk perception, and practice of pregnant women towards COVID-19 were statistically significant with generalized anxiety disorder with a *p*-value of ≤0.2. After applying multivariate logistic regression, the number of alive children, attitude, and risk perception of the pregnant women remained significantly associated with a generalized anxiety disorder at a *p*-value < 0.05.

The current study revealed that the odds of GAD among pregnant women who had less than three children was 1.53 times that of pregnant women who had greater than three children (AOR: 1.53; 95% CI: (1.11–2.13). Similarly, the odds of generalized anxiety disorder among pregnant women who had an unfavorable attitude about COVID was 1.47 times that of pregnant women who had a favorable attitude (AOR: 1.47; 95% CI: (1.07–2.02). Finally, the study revealed that pregnant women with a high-risk perception of COVID were 1.86 times more likely to develop a generalized anxiety disorder than their counterparts (AOR: 1.86; 95% CI: 1.34–2.57 (Table 3).

**Table 3:** Factors associated with generalized anxiety disorder among pregnant women attending ANC during COVID-19 pandemic at public health facilities of East Gojjam zone, Amhara region, Ethiopia, 2020. (*n* = 806).

**Variables**	**Anxiety level**		**COR (95%)**	***P*–value**	**AOR (95%)**	***P*–value**
	**No**	**Yes**				
**Residence**
Rural	223	157	1		1	
Urban	231	195	1.20 (0.91–1.59)	0.20	1.04 (0.72–1.48)	0.85
**Educational level**
No formal education	208	135	1		1	
Primary	99	71	1.11 (0.76–1.61)	0.60	1.02 (0.68–1.52)	0.52
Secondary	53	52	1.51 (0.97–2.35)	0.65	1.41 (0.86–2.33)	0.93
College and above^*^	94	94	1.54 (1.08–2.21)	0.18	1.25 (0.79–1.97)	0.18
**Number of alive children**
<3	297	255	1.39 (1.03–1.88)	0.03	1.53 (1.11–2.13)	0.01^*^
≥3	157	97	1		1	
**Attitude**
Favorable	214	198	1	0.00	1	
Unfavorable	138	256	2.01 (1.51–2.66)		1.47 (1.07–2.02)	0.02^*^
**Knowledge**
Adequate	219	197	1.36 (1.03–1.80)	0.03	1.20 (0.86–1.68)	0.27
Inadequate	235	155			1	
**Risk perception**
High	135	165	2.09 (1.56–2.79)	0.00	1.86 (1.34–2.57)	0.00^*^
Low	319	187	1		1	
**Practice**
Good	181	173	1.46 (1.10–1.93)	0.01	1.289 (0.95–1.75)	0.11
Poor	273	179	1		1	

***NB**, **1**, Reference category*;

* *Significance at a p-value < 0.05*.

## Discussion

The COVID-19 pandemic-induced disruptions in routine healthcare services, especially in the developing countries with weaker health systems, can threaten global progress toward reducing maternal and child morbidity and mortality, disproportionately worse mental health, and leads to adverse birth outcomes.

The current study reported that 43.7% of pregnant women experienced GAD during the COVID pandemic. The finding is higher than a study in Ethiopia (10.4%) done before the COVID-19 pandemic era (26). This is due to the unique stressor COVID-19 pandemic (8) is associated with an increased prevalence of anxiety in pregnant women during the pandemic (16). According to a study in China, anxiety prevalence among pregnant women was also higher during the pandemic than before it (27).

The finding of the current study was relatively higher than previous studies conducted in Ethiopia during the COVID-19 pandemic (32.2%) (9), Nigeria (37.5%) (28), and China (36.77%) (29). The finding is significantly higher than studies from the Southern Ethiopia (10.04%) (26), South Africa (23%) (18), Tanzanian (25%) (30), China (6.8%) (31), and (11.18%) (27), Pakistan (20.4%) (32), Iran (21%) (15), Brazil (26.8%) (19), and Indonesia (29%) (33). On the other hand, the finding of the current study was lower than studies in Taiwan (52.1%) (34), India (55.7%) (35), Canada (57%) (8), and Turkey (64.5%) (36). Time differences in studies and socioeconomic and cultural characteristics differences of participants contribute to the discrepancy. The discrepancy could be due to variations in measurement tools, scales, and point cutoffs used between studies. The difference in the quality of prenatal services, such as reducing waiting time for prenatal services to avoid crowded environments between health facilities, may also contribute to the discrepancy. In Africa, including Ethiopia, a high degree of mental health disorders among pregnant women may occur during the COVID-19 pandemic due to weak health systems, poor-mental health policies and infrastructure, high-poverty rates, and unreliable maternal care (20). In addition, the lack of insurance and poor counseling regarding adverse fetomaternal outcomes in developing countries such as (Ethiopia) contribute to the rising prevalence of GAD.

The odds of experiencing GAD among pregnant women with less than three children were 1.53 times that of pregnant women with greater than three children. Studies in Turkey (37), China (29, 38), and Canada (8) support this evidence, in which pregnant women with a lower level of parity had a higher prevalence of anxiety. A higher occurrence of GAD in this study was attributed to major study participants of primigravida (330) 40.9%; this group was more sensitive and could easily magnify pregnancy symptoms such as nausea and vomiting into a more severe illness, which increased the prevalence of anxiety. Fear of losing the first pregnancy and physiological changes make primigravida women more prone to develop GAD than women who have had previous pregnancy and birthing experiences. Furthermore, little parenting experience and lack of confidence in becoming mothers contribute to the high prevalence of GAD in this group of pregnant women.

The study revealed that pregnant women who had unfavorable attitude were 1.47 times higher risk of experiencing GAD than compared with counterparts. The evidence is congruent with a study in China (39). The possible explanation may be the fear of being infected with COVID-19 among pregnant women due to the insecurities and excessive worries. Exaggerated beliefs about the occurrence of poor fetomaternal outcomes such as preterm labor, miscarriage, and congenital anomalies despite available evidence (40) may contribute to an increased incidence of GAD among pregnant women with a negative attitude. A negative attitude such as stigmatization and being quarantined related to being infected with COVID-19 may contribute to the increased prevalence of anxiety. Fear of blame, guilt, and discrimination due to being infected increases the likelihood of anxiety in pregnant women with a negative attitude.

The study also found that pregnant women with a high-risk perception of COVID-19 were 1.86 times more likely than their counterparts to experience GAD. This evidence is supported by a study in China, as maternal anxiety was strongly associated with risk perception (41). The higher the risk perception, the more anxiety level occurs among pregnant women. The reason may be due to excessive concern about the health of their fetus and family member. Feeling insecure about the risk of getting COVID-19 while visiting health facilities, as pregnancy is a time of pregnant women visits health facilities more frequently to obtain maternal health services, could contribute to the anxiety during pregnancy. The impact of COVID-19 with a high level of risk perception on their pregnancy among pregnant women through changing visiting schedules and reduced activities may also be associated with increased antenatal anxiety.

### Limitations of the Study

The study attempted to ensure the representativeness of the findings. The cross-sectional nature of the study limits the cause and effect determination variables. The study was also prone to social desirability, recall, and interviewer bias. As COVID-19 pandemic results in increased anxiety level, we could not differentiate the contribution of pregnancy and the COVID-19 pandemic in this high level of GAD.

## Conclusion

The prevalence of GAD during the COVID-19 pandemic in the study area was high. Identifying factors affecting GAD in pregnant women is essential in designing effective strategies to manage mental health during the perinatal period. Less than 3 three children, having a negative attitude, and having a high-risk perception regarding the COVID-19 pandemic were factors associated with GAD. Therefore, obstetric caregivers and other stakeholders should help pregnant women to improve their risk perception and attitude by delivering appropriate information. Integrating anxiety screening programs into antenatal care should be considered to reduce GAD and its complications.

## Data Availability Statement

The original contributions presented in the study are included in the article/Supplementary Material, further inquiries can be directed to the corresponding author.

## Ethics Statement

The studies involving human participants were reviewed and approved by Ethical approval was sought from ethical review of the College of Health Science, Debre Markos University (Ref. No:-HSC/R/C/Ser/Co/214/11/13/). All study participants gave written informed consent in accordance with the declaration of Helsinki. The patients/participants provided their written informed consent to participate in this study.

## Author Contributions

KB and AA conceptualized the study. KB, AA, HA, and Tw contributed to the design of the study, data interpretation, and analysis. KB wrote the original manuscript draft. All the authors critically revised the manuscript and approved the summited version.

## Funding

This study was funded by the Debre Markos University, College of Health Science (HSC/325/20/13).

## Conflict of Interest

The authors declare that the research was conducted in the absence of any commercial or financial relationships that could be construed as a potential conflict of interest.

## Publisher's Note

All claims expressed in this article are solely those of the authors and do not necessarily represent those of their affiliated organizations, or those of the publisher, the editors and the reviewers. Any product that may be evaluated in this article, or claim that may be made by its manufacturer, is not guaranteed or endorsed by the publisher.
